# Effects of multistrain *Bifidobacteria* and *Lactobacillus* probiotics on HMO compositions after supplementation to pregnant women at threatening preterm delivery: *design of the randomized clinical PROMO trial*

**DOI:** 10.1186/s40348-024-00179-5

**Published:** 2024-08-01

**Authors:** A. Welp, E. Laser, K. Seeger, A. Haiß, K. Hanke, K. Faust, G. Stichtenoth, C. Fortmann-Grote, J. Pagel, J. Rupp, W. Göpel, M. Gembicki, JL. Scharf, A. Rody, E. Herting, C. Härtel, I. Fortmann

**Affiliations:** 1 Department of Gynecology and Obstetrics, University Hospital of Lübeck, Lübeck, Germany; 2 Department of Pediatrics, University Hospital of Lübeck, Lübeck, Germany; 3 Institute of Chemistry and Metabolomics, University of Lübeck, Lübeck, Germany; 4 Department of Microbial Population Biology, Max Planck Institute for Evolutionary Biology, Plön, Germany; 5 Department of Pediatrics, University Hospital of Hamburg-Eppendorf, Hamburg, Germany; 6 German Center for Infection Research, Lübeck, Germany; 7 Institute for Infectious Diseases and Microbiology, University of Lübeck, Lübeck, Germany; 8 Department of Pediatrics, University of Würzburg, Würzburg, Germany

**Keywords:** Probiotics, Microbiome, Preterm birth, Human milk oligosaccharides, Bifidobacteria, Entero-mammary pathway

## Abstract

**Background:**

As an indigestible component of human breast milk, Human Milk Oligosaccharides (HMOs) play an important role as a substrate for the establishing microbiome of the newborn. They have further been shown to have beneficial effects on the immune system, lung and brain development. For preterm infants HMO composition of human breast milk may be of particular relevance since the establishment of a healthy microbiome is challenged by multiple disruptive factors associated with preterm birth, such as cesarean section, hospital environment and perinatal antibiotic exposure. In a previous study it has been proposed that maternal probiotic supplementation during late stages of pregnancy may change the HMO composition in human milk. However, there is currently no study on pregnancies which are threatened to preterm birth. Furthermore, HMO composition has not been investigated in association with clinically relevant outcomes of vulnerable infants including inflammation-mediated diseases such as sepsis, necrotizing enterocolitis (NEC) or chronic lung disease.

**Main body:**

A randomized controlled intervention study (PROMO = probiotics for human milk oligosaccharides) has been designed to analyze changes in HMO composition of human breast milk after supplementation of probiotics (*Lactobacillus acidophilus*,* Bifidobacterium lactis* and *Bifidobacterium infantis*) in pregnancies at risk for preterm birth. The primary endpoint is HMO composition of 3-fucosyllactose and 3’-sialyllactose in expressed breast milk. We estimate that probiotic intervention will increase these two HMO levels by 50% according to the standardized mean difference between treatment and control groups. As secondary outcomes we will measure preterm infants’ clinical outcomes (preterm birth, sepsis, weight gain growth, gastrointestinal complications) and effects on microbiome composition in the rectovaginal tract of mothers at delivery and in the gut of term and preterm infants by sequencing at high genomic resolution. Therefore, we will longitudinally collect bio samples in the first 4 weeks after birth as well as in follow-up investigations at 3 months, one year, and five years of age.

**Conclusions:**

We estimate that probiotic intervention will increase these two HMO levels by 50% according to the standardized mean difference between treatment and control groups. The PROMO study will gain insight into the microbiome-HMO interaction at the fetomaternal interface and its consequences for duration of pregnancy and outcome of infants.

## Background and literature research

Human Milk Oligosaccharides (HMOs) are the third largest component of human milk [2, 3]. Reported concentrations range from 5-15 g/l in mature milk up to 20 g/l in colostrum [2, 4]. More than 200 different HMOs have been identified although < 20 of them account for > 90% of total content [5]. HMO concentrations differ depending on maternal factors (age, parity, diet, ethnicity) [6], lactation period, length of gestation and mother’s genetic secretor status [4]. Glycosyltransferases synthesize five monosaccharides (ß-D-galactose(Gal), ß-D-glucose(Glc), ß-*N*-acetylglucosamine(Glc*N*Ac), α-L-Fucose(Fuc) and α-D-*N*-acetylneuraminic acid (Sia)) into the different HMOs [2, 5] in the Golgi apparatus of the mammary gland cells. The concentrations of HMOs in human milk vary greatly depending on the maternal expression of the secretor gene (FUT2) and the Lewis gene (FUT3) [7, 8]. Dominant (Se) and recessive (se) alleles are differentiated and mothers can be divided in ”secretors” and “non-secretors” with and without at least one dominant allele [9–11]. Hence, the total amount of HMOs in ”non-secretors” women’s milk is significantly lower compared to that of ”secretors” [12].

HMOs exert their prebiotic effect on the microbiome in the colon [13], where they are consumed by certain groups of the microbiota such as *Bifidobacteria*, strengthen their abundance, improve the mucosal barrier and unfold immunomodulatory effects [14, 15]. They may operate as antimicrobials and prevent adhesion of pathogens by entrapping specific receptors [5]. Potential positive effects on neurocognitive development have been described, however the exact mechanisms are yet unknown [14, 16]. While HMOs form the nutritional basis for specific strains of *Bifidobacteria* [13, 17–19], most pathogenic Enterobacteriaceae are unable to utilize HMOs [20]. By favoring *Bifidobacterium. infantis* in the gastrointestinal microbial community, HMOs support immunological maturation, limit excessive inflammation and intestinal permeability. In terms of clinical outcomes, the provision of breast milk with HMOs has been associated with a decreased risk for infections, improved growth and neurodevelopmental outcomes [21, 22]. Beneficial modulatory effects on the microbiome – immunity coevolution during the first months of life [15] may explain associated risk reductions for atopic diseases and asthma [23]. In preterm infants, HMOs may prevent gut dysbiosis [24, 25] and high concentrations of specific oligosaccharides (disialyllacto-*N*-tetraose) are associated with a lower risk for necrotizing enterocolitis (NEC) [26, 27]. However, research on clinical outcomes in this study population is very limited even though preterm infants are particularly vulnerable to microbiome disturbances, sustained inflammation [28] and associated adverse outcomes [29].

Recently, first approaches to optimize the composition of HMOs in human milk have been undertaken. In a study with infants at term, Seppo et al. [1] demonstrated changes in HMO composition in breast milk after supplementation of probiotics during pregnancy, e.g. increased levels of 3-fucosyllactose.

Here, we report on the methodology of a first randomized controlled intervention study with the PICO question, whether in the **P**opulation of pregnant women > 20 + 0 weeks of gestation at risk for preterm birth between 22 + 0 and 36 + 6 weeks of gestation the **I**ntervention of daily intake of *Lactobacillus acidophilus*,* Bifidobacterium animals* subsp. *lactis and Bifidobacterium. infantis* as compared to **C**ontrol (no probiotic intake) changes the **O**utcome HMO concentration and composition in human breast milk. Specifically, we hypothesize 50% increased amounts of 3-fucosyllactose and 3’-sialyllactose after supplementation [1]. Further hypotheses are that the supplementation of *Lactobacillus acidophilus* and *Bifidobacterium animalis* subsp. *lactis and Bifidobacterium. infantis* probiotics during pregnancy increases.

1. The number of several prebiotic HMOs (e.g. 3-fucosyllactose and 3’-sialyllactose) in breast milk.
2. The abundance of *Lactobacillus* and *Bifidobacteria* in women’s vaginal flora.
3. The abundance of *Lactobacillus* and *Bifidobacteria* in the neonatal microbiome of preterm infants.

Probiotics have been supplemented in several studies to pregnant women defining various hypotheses and outcomes. In preparation of our trial, we performed an extensive review of the scientific literature including (a) randomized placebo-controlled trials that have been (b) recently published (between 2010 and 2022), that (c) supplemented groups of *Bifidobacteria* spp. and/or *Lactobacillus* spp. to (d) pregnant women with an (e) minimum sample size of *n* = 30 women supplemented. We searched PubMed using the terms “probiotics” AND/OR “pregnancy” OR “probiotic supplementation” AND (“Bifidobacteria” AND/OR “Lactobacillus”) or “probiotics“ AND “preterm risk” OR “maternal supplementation” AND “bacterial diversity” OR “probiotics” AND “pregnancy” AND ”allergies” OR “probiotics” AND “pregnancy” AND “group B Streptococcus” OR “probiotics” AND “pregnancy” AND (“ bacteria AND/OR “bacterial diversity”) OR “probiotics” AND “pregnancy” AND “ obesity” .

142 publications were reviewed, 15 clinical trials were finally included considering above mentioned criteria (a-e).

In Table 1. we present the results of our literature research stratified to study population, probiotic strain used, intervention period, outcomes measured and study results. The studies report on several maternal outcomes including gestational diabetes (GDM), inflammatory markers and characteristics of the vaginal, gut or breast milk microbiome. Halkjaer et al. [30] supplemented 50 obese pregnant women with *Bifidobacteria spp.* and *Lactobacillus spp.* No significant changes in frequency of GDM (gestational diabetes mellitus), maternal HbA_1_C or infant birth weight were found. However, the gastrointestinal microbiome of supplemented pregnant women was characterized by an increased α- diversity when compared to untreated controls. Unlike these results, Luoto et al. [31] reported a reduced risk of GDM (OR = 0.27 (95% CI 0.11, 0.62); *P* = 0.002) after probiotic supplementation (*Bifidobacteria spp.* and *Lactobacillus spp.)* of 256 women throughout all trimesters of pregnancy. Study results of probiotic effects on inflammatory markers during pregnancy and perinatal period are inconclusive. Vitali et al. [32] reported on significant decreases of serum anti-inflammatory cytokines such as IL-10 (interleukin) and IL-4 in 30 women. In mothers-to-be with GDM, decreased levels of C-reactive protein were found after supplementation of *Bifidobacteria spp.* and *Lactobacillus spp.* [33] However, Dewanto et al. [34] could not reveal changes of IL-8 in breast milk, nor in fecal α-1-antitrypsin or calprotectin levels in neonatal stool.

**Fig. 1:**
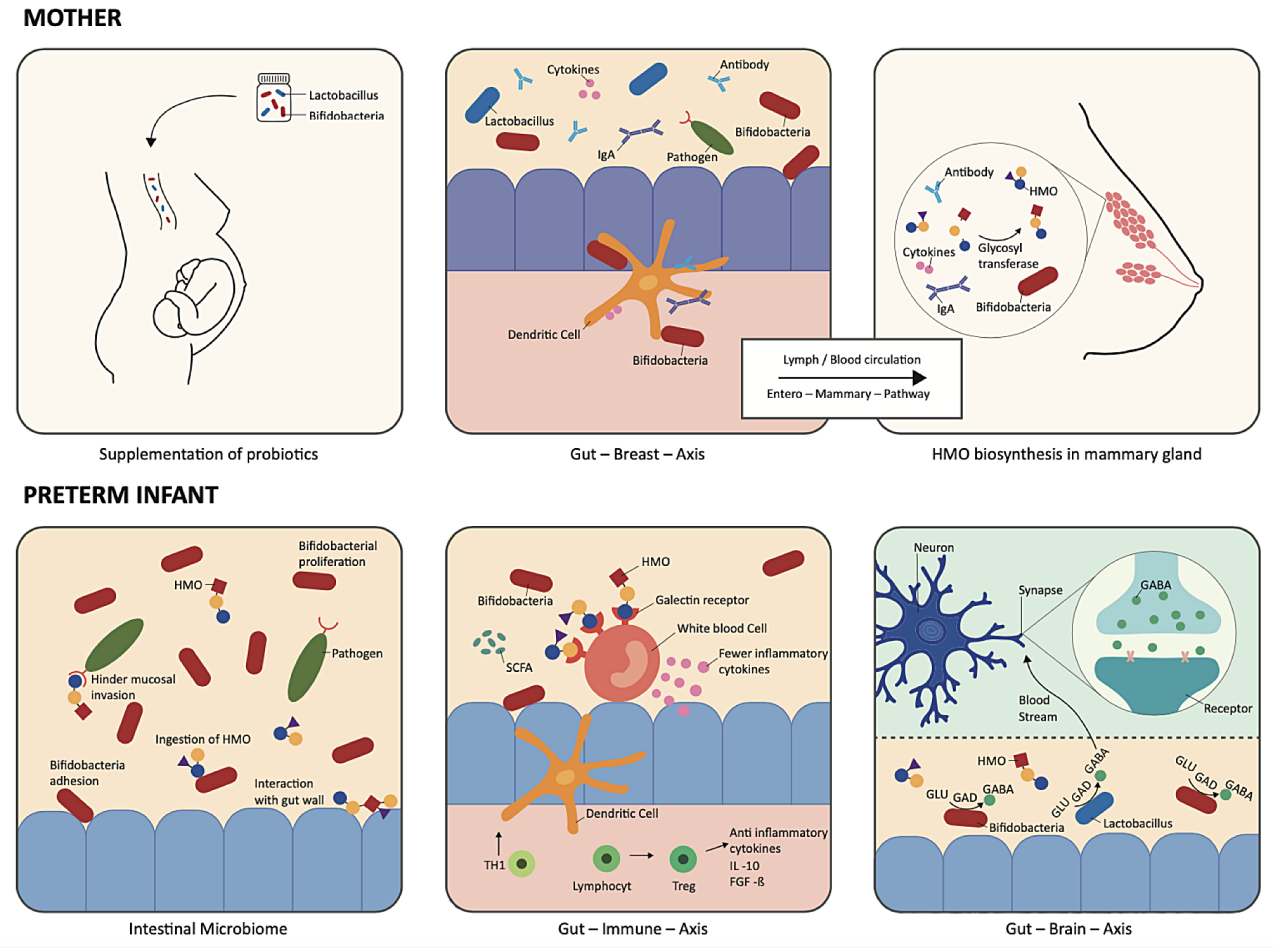
Effects in mother and preterm infant after probiotic supplementation. *Legend* FGF-ß Fibroblast growth factor beta, GABA Gamma-aminobutyric acid, GAD Gamma decarboxylase, GLU Glutamate, HMO Human milk oligosaccharide, IL Interleukin, SCFA Short chain fatty acids, T_H_-1 T-helper lymphocyte, Treg T regulatory lymphocyte

Several studies examined probiotic effects in microbiome characteristics of different niches (rectovaginal, gastrointestinal, breast milk) with heterogenous results. Abundances of *Bifidobacteria spp.* and *Lactobacillus spp.* were significantly increased in colostrum and mature milk [35], but changes in diversity of maternal vaginal microbiome’s composition have not been observed [36]. Further studies showed that the abundances of group B Streptococcus and several *Candida spp.* in the vaginal microbiome were diminished after probiotic supplementation of pregnant women [37, 38]. To our best knowledge, there is only one study that reports on HMO composition in breast milk after probiotic supplementation during pregnancy [1]. Various HMOs showed a significant increase in human milk following the intervention, which was most pronounced for 3-fucosyllactose (p = 0.008) and 3’-sialyllactose (*p* = 0.006). The authors hypothesized a shift from 6’ sialylation to 3’ sialylation. These changes might be associated with increased abundance in mammary gland after probiotic supplementation through the entero-mammary-pathway (see Fig. 1). Furthermore, various studies aimed at clinical outcomes of infants whose mother’s received probiotics during pregnancy and report on microbiome alternations. However, no significant changes were seen in children’s microbiome composition and in α- and β-diversity [39]. Notably, a decreased risk for developing atopic dermatitis in the first 18 months [40] and reduced rates of positive skin prick test results were found after probiotic supplementation during pregnancy [41].

**Table 1:** Results of literature research

Study	Study population	Type	Intervention/probiotic	Control	Intervention period	Outcomes measured	Results
Gestational diabetes (GDM)							
Halkjaer et al. [30] Denmark	50 obese pregnant women	RCT	Visbiome® 450 billion CFU Lactobacillus paracasei, plantarum, acidophilus, delbruecki; B. lactis, breve Streptococcus thermophilus	placebo	14–20 weeks of gestation until delivery	GWG, GDM, maternal HbA_1_C, infant weight	no significant group differences increased α-diversity in fecal microbiota of supplemented women
Luoto et al. [31] Norway	256 pregnant women	RCT	Lactobacillus rhamnosus GG and Bifidobacterium lactis Bb12	placebo	first trimester until delivery	frequency of gestational diabetes	significantly lower frequency of GDM no significant reduction of birth weight
Inflammatory markers							
Vitali et al. [32] Italy	30 pregnant women	RCT	VSL#3 Lactobacillus acidophilus, plantarum, paracasei, delbrueckii subsp.bulgaricus Bifidobacterium longum, breve, infantis S. thermophiles	placebo	33 to 37 weeks of gestation	cytokine secretion in women’ blood	significantly lower levels of IL-4 and IL-10 in control group
Dewanto et al. [34] Indonesia	110 pregnant women	RCT	Bifidobacterium lactis animalis HNO19	placebo	third trimester until 3 months after birth	IL-8 in breast milk, urine IFABP, faecal α-1-antytripsin (AAT) and calprotectin at birth and after 3 months	no significant alterations found between probiotic group and control group
Badehnoosh et al. [33] Iran	60 women with GDM	RCT	Lactobacillus acidophilus, lactobacillus casei, bifidobacterium bifidum.	placebo	6 weeks intervention	fasting plasma glucose, c-reactive protein, plasma malondialdehyde concentration (MDA),	significantly decrease of in fasting plasma glucose, c-reactive protein and in MDA increase of total antioxidative capacity
Mastromarino et al. [35] Italy	67 healthy pregnant women	RCT	VSL#3 Lactobacillus acidophilus, plantarum, paracasei, delbrueckii subsp.bulgaricus Bifidobacterium longum, breve, infantis S. thermophiles	placebo	36 weeks of gestation until delivery	amount Lactobacilli and Bifidobacteria in colostrum and mature milk	Significant increase in abundances of probiotic strains in probiotic group regardless the mode of delivery
Dotterud et al. [39] Norway	243 pregnant women	RCT	Lactobacillus rhamnosus GG, L acidophilus La-5, and Bifidobacterium animalis subsp. Lactis Bb-12	placebo	36 weeks of gestation up to three months postnatally	bacterial classes and genera, α- and β-diversity in children´s microbiome	no alternations in microbiome composition, α- and β-diversity
Yang et al. [36] Canada	86 pregnant women with increased nugent score	RCT	Lactobacillus rhamnosus GR-1 and Lactobacillus reuteri RC-14	placebo	12 weeks intervention starting at 13 weeks of gestation	Shannon diversity index at 13, 28 and 35 weeks of gestation in vaginal microbiome	no differences between groups
Ho et al. [37] Taiwan	110 GBS positive pregnant women	RCT	Lactobacillus rhamnosus GR-1 and Lactobacillus reuteri RC-14	placebo	21.1 ± 5 days until delivery	maternal GBS colonization	Significant decrease in GBS colonization rate in probiotic group
Ang et al. [38] Malaysia	78 pregnant women with vaginal candidiasis	RCT	Lactiplantibacillus plantarum helveticus; Lacticaseibacillus rhamnosus Lacticaseibacillus paracasei, Limosilactobacillus fermentum Lactobacillus delbrueckii subsp. Lactis	placebo	eight weeks from 14–32 weeks of gestation	abundance of Candida albicans, glabrata and Lactobacillus cripatus and jensenii in vaginal microbiome	significantly decreased abundance of C. albicans and glabrata, increased abundance of L. crispatus & L. jensenii
Atopic diseases and allergies (infants)							
Allen et al. [41] UK	554 pregnant women	RCT	Lactobacillus salivarius CUL61, 6.25 × 109 CFU/day; L. paracasei CUL08; Bifidobacterium animalis ssp. Lactis CUL34, Bifidobacterium bifidum; 1.25 ×109 CFU/day/each strain for four weeks	placebo	36 weeks of gestation to six months after delivery	Positive SPTs to food allergens (cow’s milk and egg proteins) at either 6 months or two years	Significant decrease in the rate of SPT + to cow milk and eggs in probiotic group after six months; no differences after two years.
Enomoto et al. [40] Japan	130 pregnant women	RCT	Bifidobacterium breve M-16V and Bifidobacterium longum BB536	placebo	1 month prior to delivery, postnatally for 6 months	allergic symptoms at 4, 10 and 18 months of age	significantly lower risk of developing atopic dermatitis (AD) eczema during the first 18 months of life in the probiotic group
HMOs-composition in breast milk							
Seppo et al. [1] Finland	81 colostrum samples from 1223 pregnant women	RCT	Lactobacillus rhamnosus GG, LC705, Bifidobacterium breve Bb99, Propionibacterium freudenreichii subspecies shermanii JS	placebo	36 weeks of gestation until delivery	changes in composition of human milk oligosaccharides in breast milk	significantly higher concentrations of 3-fucosyllactose and 3’-sialyllactose

*Legend GWG* gestational weight gain, *GDM* gestational diabetes mellitus, *RCT* randomized controlled trial, *GBS* group B streptococcus, *SPT* skin prick test, *CFU* colony forming unit, *IL* interleukin, *MDA* malondialdehyde, *HbA*_*1*_*C* glycolyzed hemoglobin, *C* Candida, *L* Lactobacillus

#Results refer to the group with probiotic supplementation if not otherwise indicated

Figure 1. Schematic illustration of the *entero – mammary – (neonatal) gut – pathway* which is hypothesized to be stimulated by the supplementation of probiotics during pregnancy. After maternal probiotic intake an increased abundance of *Bifidobacteria spp.* and *Lactobacillus spp.* may trigger the gut – breast axis in which probiotic bacteria, antibodies, IgA and cytokines are translocated from the gut to the mammary glands secretory cells via dendritic cells (DC) through lymph / blood circulation [42]. DCs sample commensal bacteria and immune factors by penetrating the gut mucosa via self-expressed tight junctions. An increased abundance of probiotic strains in the mammary gland cells may trigger the synthesis of HMOs as a nutritional source for *Bifidobacteria spp*., whereas the distinct mechanisms are yet unknown. Consequences for the neonatal gut microbiome include an increased abundance of probiotic bacteria which exert anti-inflammatory and infection-preventive effects by strengthening the mucosal barrier and elimination of pathogens. An improved supply of breast milk with prebiotic HMOs further supports a *Bifidobacteria* – dominated gut microbiome [43]. Beneficial effects for the neonatal “gut-immune-axis” include inhibitory effects on *Campylobacter spp.)* to the intestinal mucosa. Ancillary HMO related (2’- and 3’-Fucosyllactose) stimulation of T_H_1- lymphocytes and induction of anti-inflammatory cytokines such as IL-10 or FGF-ß distributed by regulatory T-lymphocytes is expected, alongside with increased expression of dendritic cells and maturation of naïve CD4^+^ T-lymphocytes to regulatory T-lymphocytes enhanced by certain *Bifidobacteria spp.* [44]. Short chain fatty acids (SCFA) modulate the immune tolerance by decreasing the awareness of macrophages to commensal [45, 46]. The neonatal “gut-brain-axis” is influenced by neurotransmitters such as GABA produced by *Bifidobacteria spp.* and *Lactobacillus spp.* [47]. GABA is translocated from gut to brain via blood circulation and regulates the production of the neurotransmitters like serotonin, acetylcholine and dopamine. Dysfunctions in the GABAergic system may contribute to stress associated disorders or encourage memory and cognitive impairment. [47]. Sialylated HMOs (e.g. 3’- Sialyllactose) may enhance learning, memory and language function [48], while high concentrations of 2’-Fucosyllactose in breast fed infants were found to be associated with improved motoric skills at the age of 24 months [49, 50].

## Study design and methodology

### Screening and study participants

From January 2024 to January 2026 all women presenting with risk for preterm birth (e.g. cervical shortening, PPROM, vaginal bleeding, preeclampsia, HELLP syndrome) are informed about the study and asked to participate after giving their written informed consent. The women will be given probiotics from the day that they are identified with a risk of imminent preterm birth and decide to participate in this study until the delivery of the infant. We will include approximately 130 women from 20 + 0 to 36 + 6 weeks of pregnancy. The study population will be randomly divided into two groups consisting of (1) women taking probiotics during pregnancy and (2) women without probiotic supplementation. This study is registered at the German Registry for clinical studies (DRKS), DRKS00033539.

### In- and exclusion criteria

Pregnant women of any ethnic background between 20 + 0 and 36 + 6 weeks of pregnancy who are at risk for preterm birth are included. The underlying condition (e.g. PPROM, cervical shortening, bleeding, preeclampsia, HELLP syndrome) is not decisive to inclusion. Participating patients will be included after they have been informed about the study procedures, intervention, associated responsibilities and when they have given written informed consent. Women with outpatient monitoring are also applicable for participation. Since the date of birth cannot be predicted, the study concept inevitably includes full-term children – mother pairs to be included. Pregnant women under the age of 18 years and women, who do not plan to deliver their infant at our site will be excluded primarily, because in these cases collecting the samples postpartum is not feasible. Women who decline to feed their infant with breast milk or without written consent will also be excluded.

### Probiotic formula

The probiotic formulation is Bactiol^®^ Infantis consisting of *Bifidobacterium longum* subsp. *Infantis*,* B. animalis* subsp. *Lactis (BB-12) and Lactobacillus acidophilus (La-5)*. These strains have been commonly used in former studies supplementing preterm infants [51–53]. The women are provided a single dose capsule once daily. Each dose contains 4.5 × 10^9^ colony forming units of the bacteria mixture. We chose this particular formula because it has been previously used in clinical trials. The multistrain probiotics raised no safety concerns in large observational studies in preterm infants and the PRIMAL trial [52] and is approved for use in pregnancy. Several strains of lactobacilli are also able to utilize HMOs, but with less capacity compared to Bifidobacteria [60]. Lactobacillus acidophilus has the ability to hydrolyze HMOs and studies report moderate growth of Lactobacillus acidophilus (LA-5) on lacto-N-neotetraose and on lactose [61, 62].

### Intervention/Control

Study participants are randomly assigned to receive the probiotic mixture (intervention group *n* = 73) or not (control group *n* = 73). The intervention is one capsule of Bactiol^®^ Infantis per day until delivery. The proposed number of participants is calculated based on former studies (see below). A randomization sequence was created in advance to assign mother – infant pairs to a study arm. This trial is not blinded. The women and participating doctors know whether they are in the intervention or control group, and we are not using a placebo.

### Stopping rules

Any participating women may be retired from the study at any time at her own request, unbiased by reason and without facing any disadvantages of clinical support. Women who are withdrawn from the study, are not allowed to re-enter the study. The responsible doctor has the right to exclude women from the study, who deliver at another hospital and women with discontinuous intake of the probiotics in more than 30% of days until delivery. To ensure that the data is recorded according to the intention-to-treat principle, all patients should be followed up and documented after retirement from the study.

### Primary endpoint, secondary endpoints/data collection

The primary endpoint is the HMO composition (3-fucosyllactose, 3’-sialyllactose) in human breast milk after supplementation prenatally. Secondary endpoints are clinical and safety outcomes of mothers (infections, alterations in vaginal microbiome) and infants (preterm birth, sepsis, growth, gastrointestinal complications) and the effects on the microbiome composition in the rectovaginal tract of mothers at delivery and in the gut of term and preterm infants.

### Sample collection

**Fig. 2:**
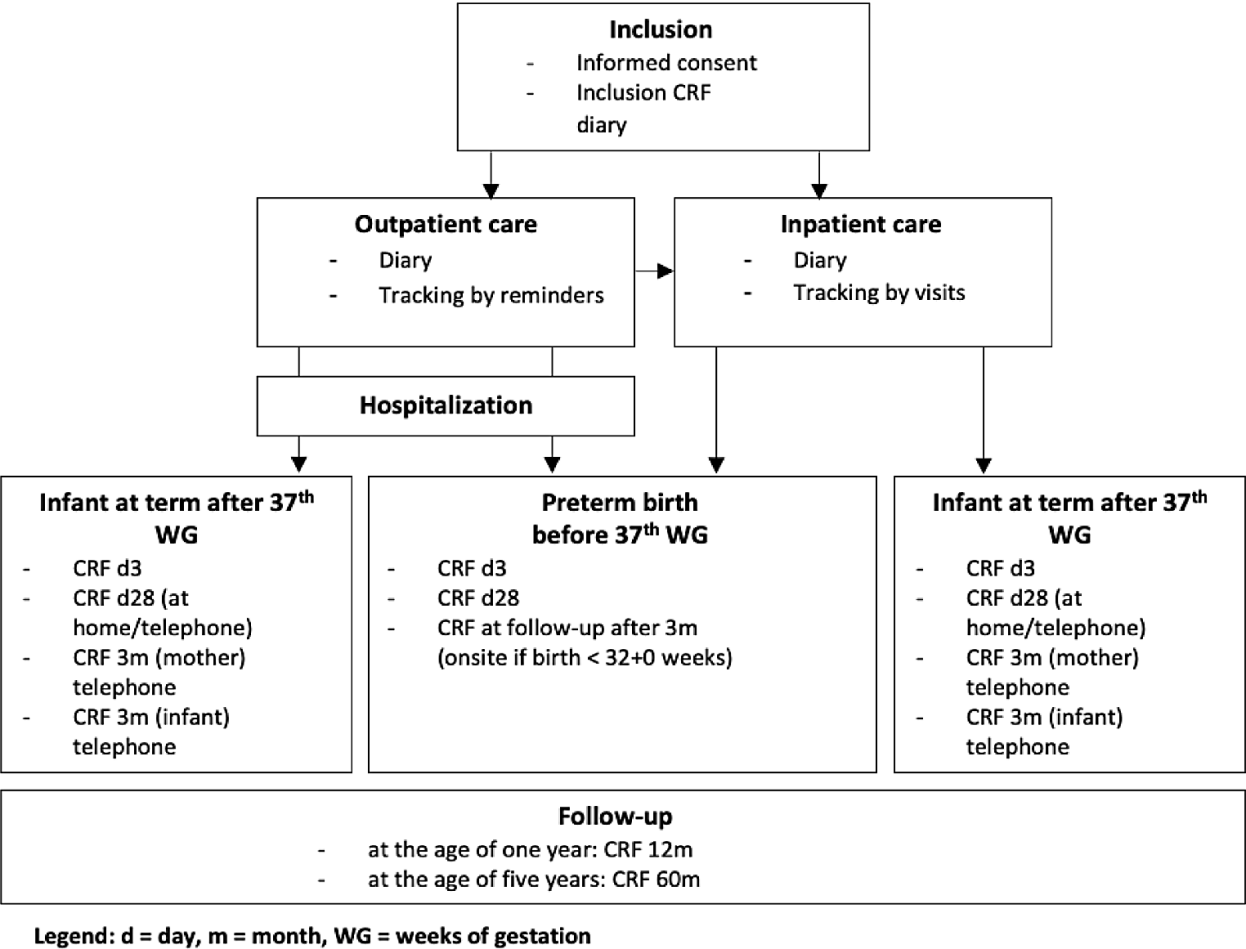
Flow chart of used CRFs

After informed consent the participants take one capsule of probiotics once daily until delivery. At the timepoint of inclusion, samples to describe the “baseline microbiome” will be collected in the form of a rectovaginal swab and a stool sample. As soon as labor begins or imminent delivery is likely, a rectovaginal swab and stool sample are taken by a midwife or obstetrician to test hypothesis 2 (increased abundance of *Lactobacillus* and *Bifidobacteria* in women’s vaginal flora due to probiotics supplementation in pregnancy). After birth samples of colostrum, breast milk and neonatal stool are gathered on day one, two, three, seven, fourteen and 28, stored and frozen at -80 °C. Depending on gestational age, stage of enteral feeding, and growth, preterm infants are fed exclusively breastmilk, donor milk, formula milk or a mixture. This will be considered in the final analysis. Additional samples (a stool sample of the infant and a breast milk sample from the mother when still breastfeeding) will be collected at the follow-up visit at three-month, 1 year and 5 years of age by study personnel. A standardized questionnaire about former parents’ history, infections, allergies, postpartum course, provided by the families in the first week, completed by case report forms at day 3, day 28 and at three months, one year and five-year follow-up (see Fig. 2).

### Data collection

Standardized Case Report Forms (CRFs) are used to record information from the mother prenatally and postnatally from mother and infant. At the time of inclusion (CRF inclusion), closely after birth (CRF day 3), one month after birth (CRF day 28), at three months CRF 3 m (mother + infant), one- and five-year follow- up (CRF 12 and 60 m) (see Fig. 2).

Data collected at the follow-ups will be used to measure long-term outcomes (allergies, incidence of infections, re-admissions to the hospital). The CRFs are filled in manually by the study team (neonatologist, doctoral student and study nurse). Additionally, the women will be asked to keep a diary handed out at inclusion to document regularity of probiotic intake, infections, antibiotic therapy, allergies and possible side effects during the intake of probiotics. The inclusion-CRF collects information of former participant’s medical history e.g. preexistent diseases, the course of pregnancy, former pregnancies and allergies. The follow up CRFs collect data from both, mothers and infants. CRF day 3 includes information about the delivery mode, reason for delivery, infections and risk factors for microbiome dysbiosis. In CRF day 28 information about complications in puerperium, growth parameters and feeding of the infant is collected. This data will be collected via telephone interview. In case of birth before completed 32 weeks follow-up examination will take place onsite. At follow-up information on both, mother and infant will be recorded via CRFs.

### HMO-assessment

Analysis of HMOs amount and composition will be performed at the Institute of Chemistry and Metabolomics of the University of Lübeck, Germany, by nuclear magnetic resonance (NMR) spectroscopy). To this end, samples of breast milk will be analyzed according to published protocols [54]. Briefly, proteins and lipids will be removed by ultrafiltration and after buffer addition to the filtrate, NMR spectra will be acquired. NMR spectra will be analyzed and compared with published data [54, 55] to (i) determine the secretor status of the woman and if the woman is Lewis positive or negative and to (ii) investigate if the concentration of HMOs in breast milk change with probiotic supplementation.

### Microbiome sequencing

To address hypothesis 3 (increased abundance of several bacteria in neonatal microbiome) 16s rRNA microbiome analyses will be performed at the Clinic for Infectiology and Microbiology at the University of Lübeck, Germany [53, 56]. To amplify partial sequences of 16 S rRNA gene linker and indices-containing primers targeting V3/V4 hypervariable regions of 16 S rRNA [29] will be used. Polymerase chain reaction will be performed starting with 98 °C for 30 s, followed with 30 cycles with 98 °C for 9 s, 55 °C for 60 s and 72 °C for 90 s finishing with 72 °C for ten minutes. Storage of amplicons at -20 °C until further processing is planned. Agarose gel electrophoresis will be used to estimate the amplicons concentrations. GeneRuler 100 bp DNA Ladder (Thermo Fischer Scientific, Waltham, USA) will be used as reference. Sequencing will be performed using MiSeq^®^ platform (Illumina^®^, San Diego, California, USA) and MiSeq^®^ reagent Kit V3 for 600 cycles. PhiX library will be set as positive control. To ensure absence of reagents contamination negative extraction controls will be integrated, too.

### Estimated sample size and power calculations

Based on the publication of Seppo et al. [1] who supplemented probiotics in pregnancies > 36th weeks calculations of sample sizes were performed.

**Table 2:** Calculation minimal sample size based on data from Seppo et al. 2019 [1]

Human milk oligosaccharids	Supplementation	No supplementation	Minimal sample size
	mean [SD]	mean [SD]	*n*
3-fucosyllactose (µmol/mL)	413 [164]	312 [232]	73
3´-sialyllactose (µmol/mL)	833 [679]	516 [378]	55
difucosyllacto-*N*-hexaose (µmol/mL)	62.0 [43.3]	93.7 [47.7]	38
lacto-*N*-tetraose (µmol/mL)	509 [339]	861 [563]	32
lacto-*N*-fucopentaose I (µmol/mL)	2000 [585]	2450 [836]	47
6´-sialyllactose (µmol/mL)	472 [159]	567 [179]	58

*Legend SD* standard deviation; *n* estimated sample size

Table 2. lists HMO measurement means and standard deviations taken from Seppo et al. [1] for six different HMO subgroups. Column titled “n” lists minimal sample size numbers inferred via balanced Mann-Whitney U test. Calculations were performed with the R package ’pwrss’ [Bulus, M. (2023). pwrss: Statistical Power and Sample Size Calculation Tools. R package version 0.3.1.]. The significance threshold (acceptable type I error rate) was set to alpha = 0.05 and predictive power (acceptable Type II error rate) was set to 0.8. Dropout rate was considered to be 10%. Whereas sample size numbers in Seppo et al. [1] are unbalanced between treatment group and control group (50 vs. 31), we assumed balanced conditions in our analysis to minimize the total number of required samples. Based on these calculations we include 73 women taking the probiotics ( ≙ verum group) during pregnancy and 73 women without treatment ( ≙ control group). We choose the maximal calculated number n (Table 2.) as minimal sample size and add a 25% risk of drop out.

### Statistical analysis

Subgroup analysis of previous medical history exposure to antibiotics, infections in pregnancy and indication for delivery will be performed on an exploratory basis. Adjustments for recall rate (diary, survey data) will be performed, we aim for a recall-rate of > 75%.

Statistics will be performed with R version 4.2 or higher. For graphs Graphpad prism version 10.1.2. will be used. Details of the statistical analysis will be fixed in a statistical analysis plan, which will be finalized by the trial statistician before inclusion of the last patient. The duration of probiotic intake will be taken into account in the final analysis. We will adjust our data for the different intervention timeframes using matched pair and multivariate regression analyses. However, the duration of pregnancy-accompanied probiotic therapies that were necessary to achieve the investigated outcome effects in the various studies performed (see Table 1.) have not yet been investigated. Consequently, in the case of observed effects of probiotic therapy on the HMO concentration, we will also determine the necessary duration of supplementation as a result of this study.

### Data handling and safety

All data is stored pseudonymized. Data of individual participants cannot be traced back. Personal data of the patients will be saved separately for further contacts of the families. The procedures to save and store the data adhere to German data protection laws.

### Data safety monitoring board

An independent data safety monitoring board consisting of an obstetrician and neonatologist, both with broad clinical expertise and experience in scientific trials will assemble on regular basis to review data quality, study procedures, CRFs, safety and results.

### Quality insurance and safety

If any unwanted events appear during the intake of probiotic or postpartum the women are obliged to contact us immediately. The study will be conducted, data recorded according to the protocol, the standard operating procedures (SOP) of our both clinics, the Good Clinical Practice (GCP) and the connected regulations and requirements.

### Data sharing and dissemination

Authorship of resulting manuscripts will be based on guidelines of the International Committee of Medical Journal Editors. Involved parties will be informed in cases of important modifications. The results of this study will be published in peer-review journals and presented at scientific meetings, which will enable discussions with scientific community.

## Conclusion

This is a prospective randomized intervention study supplementing probiotics to women with imminent preterm birth. We propose to support HMO composition in breast milk and consequently to increase the abundance of favorable bacterial groups (primarily *Bifidobacteria* spp.) in the neonatal microbiome. The aim of this study is to improve clinical neonatal outcomes by optimizing the supply of HMOs in human breast milk in order to create improved conditions for the establishment of a healthy neonatal microbiome. Further, we aim at defining microbiome signatures of the women’s vaginal flora that are associated with probiotic intake and HMO composition. We hope to learn more about the complex coevolution of pregnant women’s gut microbiome, immune modulating components of human breast milk (e.g. lactoferrin, IgA or glycomacropeptide) and immune function of the newborn [57, 58]. The biophysiological mechanisms of this coevolution is described by the concept of an “*entero – mammary – (neonatal) gut pathway*” which not only seems to determine the supply of commensal bacteria in breast milk at the critical timeframe of neonatal microbiome establishment within the first weeks of life. Immune factors, cytokines, regulatory cells and antibodies are translocated in a similar manner to potentially affect and shape neonatal vulnerability in the “*window of opportunity*” after delivery. That the *entero – mammary – gut pathway* can be successfully targeted by probiotic supplementation of pregnant women has been previously demonstrated by a randomized placebo-controlled trial (RCT) that measured elevated concentrations of *Bifidobacteria* spp. in breast milk of treated women when compared to untreated controls [35]. Notably, various (patho)physiological elements in this context have not yet been studied in detail, including determinants of HMO synthetization in the mammary glands. However, previous studies point towards more complex translocation processes that are stimulated by probiotic supplementation during pregnancy. These processes in passive immunization and antibody translocation as well as HMO supply in expressed breast milk.

Probiotics are widely used in preterm infants as a NEC prophylaxis [51, 52] and were supplemented to pregnant women within clinical trials [1]. To identify the scientific value of this study, we performed a literature review on probiotic supplementation during pregnancy reporting on various maternal and neonatal outcome parameters. Interestingly, RCTs using *Lactobacillus acidophilus* and *Bifidobacterium animals subsp. lactis and B. infantis* probiotics not only showed a significant impact on maternal outcomes such as decreased rates of GDM [31] or reduced colonization rates with group B Streptococcus [37], but as well demonstrated significant measurable effects on neonatal characteristics such as microbiome composition, immune phenotype (cytokine secretion) and rates of atopic symptoms [40, 41]. In conclusion, however, study results are largely inconclusive across the studies performed, which might mainly be due to differences of in- and exclusion criteria, dosage of probiotic supplements and most importantly, targeted outcomes. To our best knowledge and as a result of our literature review, only one study has investigated HMO composition of human breast milk after probiotic supplementation of pregnant women. While demonstrating promising results by showing increased levels of several breast milk HMOs after birth, the study was limited to included pregnancies of > 36 weeks and infants born at term. Hence, the effects of probiotic supplementation have so far never been studied in the context of HMO breast milk composition at preterm birth. Our main hypothesis is that supplementation of *Lactobacillus acidophilus* and *Bifidobacterium animals subsp. lactis and Bifidobacterium infantis* during pregnancy will increase the abundance of HMOs at the time of preterm delivery. Based on the findings of Seppo et al. [1], we propose changes of the concentration of 3’-fucosyllactose and 3’-sialyllactose in women supplemented with probiotics when compared to non-treated controls. Our second hypothesis of vaginal microbiome signatures that are associated with probiotic intake are based on study results showing a reduction in vaginal dysbiosis and increased amounts of favorable bacteria [35, 39, 59]. As a secondary outcome we will explore signatures of the neonatal microbiome of supplemented mothers as compared with non-supplemented controls. Previous observational studies and RCTs indicate that probiotics are safe and well tolerated in an adequate clinical setting. Results of this study may have implications for the management of pregnancies at risk for preterm delivery. A stable neonatal microbiome and dysbiosis prevention may improve short and long-term outcomes of preterm infants. If this study confirms our hypotheses, placebo-controlled randomized trials will be necessary to verify the results.

## Data Availability

No datasets were generated or analysed during the current study.

## Abbreviations

C: Candida
CFU: Colony forming unit
CRF: Case report form
DNA: Desoxyribonucleic acid
FGF-ß: Fibroblast growth factor beta
GABA: Gamma-aminobutyric acid
GAD: Gamma decarboxylase
GBS: Group B streptococcus
GCP: Good clinical practise
GDM: Gestational diabetes mellitus
GLU: Glutamate
GWG: Gestational weight gain
HbA_1_C: Glycolyzed hemoglobin
HMO: Human milk oligosaccharide
IL: Interleukin
L: Lactobacillus
MDA: Malondialdehyde
NEC: Necrotizing enterocolitis
RCT: Randomized controlled trial
rRNA: Ribosomal ribonucleic acid
SCFA: Short chain fatty acids
SOP: Standard operating procedures
SPT: Skin prick test
T_H_-1: T-helper lymphocyte
Treg T: regulatory lymphocyte
